# Comparison of Azelnidipine and Trichlormethiazide in Japanese Type 2 Diabetic Patients with Hypertension: The COAT Randomized Controlled Trial

**DOI:** 10.1371/journal.pone.0125519

**Published:** 2015-05-04

**Authors:** Masahiro Takihata, Akinobu Nakamura, Yoshinobu Kondo, Satsuki Kawasaki, Mari Kimura, Yasuo Terauchi

**Affiliations:** 1 Department of Endocrinology and Metabolism, Yokohama City University School of Medicine, Yokohama, Kanagawa, Japan; 2 Department of Endocrinology and Metabolism, Chigasaki Municipal Hospital, Chigasaki, Kanagawa, Japan; 3 Department of Endocrinology and Metabolism, Shonan Fujisawa Tokushukai Hospital, Fujisawa, Kanagawa, Japan; Kurume University School of Medicine, JAPAN

## Abstract

**Objective:**

This study compared the efficacy and safety of azelnidipine with that of trichlormethiazide in Japanese type 2 diabetic patients with hypertension.

**Methods:**

In a multicenter, open-label trial, 240 patients with adequately controlled diabetes (HbA1c ≤ 7.0%) under lifestyle modification and/or administration of hypoglycemic agents and inadequately controlled hypertension (systolic blood pressure [sBP] ≥ 130 mmHg or diastolic blood pressure [dBP] ≥ 80 mmHg) who were being treated with olmesartan were enrolled. Participants were randomly assigned to an azelnidipine group or a trichlormethiazide group and were followed up for 48 weeks. Main outcome measure was the difference in the change in HbA1c levels from the baseline values at 48 weeks between these two groups.

**Results:**

Of the 240 subjects that were enrolled, 209 subjects (azelnidipine group: 103 patients, trichlormethiazide group: 106 patients) completed this trial. At 48 weeks, the following changes were observed in the azelnidipine and trichlormethiazide groups, respectively: HbA1c levels, 0.19 ± 0.52% and 0.19 ± 0.54%; sBP/dBP, -10.7 ± 9.6/-6.6 ± 6.6 mmHg and -7.1 ± 7.7/-3.3 ± 6.1 mmHg (P < 0.001 for both sBP and dBP). In both groups, dizziness (12 patients [11.7%] and 16 patients [15.1%]) and edema (16 patients [15.5%] and 7 patients [6.6%], P = 0.047) were observed during the 48-week follow-up period.

**Conclusions:**

Azelnidipine was more effective for controlling blood pressure than trichlormethiazide in Japanese type 2 diabetes patients, whereas trichlormethiazide was more effective for reducing albuminuria than azelnidipine. Both of these agents, however, similarly exacerbated glycemic control in type 2 diabetic patients with hypertension.

**Trial Registration:**

UMIN 000006081.

## Introduction

Type 2 diabetes and hypertension are commonly encountered diseases that coexist frequently. Hypertension in type 2 diabetic patients increases the risks of cardiac disease, peripheral vascular disease, stroke, retinopathy, and nephropathy [1]. Controlling not only diabetes but also hypertension and preventing their complications in type 2 diabetic patients with hypertension is thus an extremely important issue.

The American Diabetes Association (ADA), the Seventh Report of the Joint National Committee (JNC-7), the European Society of Hypertension (ESH), and the Japanese Society of Hypertension recommend the use of angiotensin-converting enzyme inhibitors (ACE inhibitors) and angiotensin receptor blockers (ARBs), together with lifestyle modifications, for type 2 diabetic patients with hypertension [2–5] because these agents decrease the risk of cardiovascular events [6–9], diabetic retinopathy [10], and diabetic nephropathy [9, 11–18]. In addition, they also delay the onset of diabetes [6, 13, 19–22]. Thus, ACE inhibitors or ARBs are recommended as the first step in the treatment of type 2 diabetic patients with hypertension.

However, in type 2 diabetic patients with hypertension that is inadequately controlled using ACE inhibitors or ARBs, the additional use of antihypertensive medications is controversial. The calcium blocker azelnidipine whose effect is no way inferior to that of amlodipine [23] and the thiazide diuretic trichlormethiazide are also recommended as a second step. Calcium blockers decrease the risk of cardiovascular events as well as ACE inhibitors or ARBs in type 2 diabetic patients with hypertension [20, 24–28] and previous study showed that a combination of olmesartan and azelnidipine improved HbA1c level significantly [29] and azelnidipine significantly decreased levels of glucose and insulin 120 min after the 75 g oral glucose tolerance test [30].

A combination of the ACE inhibitor benazepril and the calcium blocker amlodipine decreased the risk of cardiovascular events, compared with the combination of benazepril and thiazide diuretic hydrochlorothiazide [31]. On the other hand, the combination of benazepril and hydrochlorothiazide improved microalbuminuria, compared with the combination of benazepril and amlodipine [32].

Thiazide diuretics decrease the risk of cardiovascular events [20, 33–34], and a combination of the ACE inhibitor perindopril and the thiazide diuretic indapamide decreases the risk of cardiovascular events [35]. However, thiazide diuretics exacerbate glucose metabolism, lipid metabolism, hypokalemia, and hyperuricemia [20, 33, 36]. The incidences of the adverse effects of diuretics increase in a dose-dependent manner [37], with low-dose diuretics being tolerable [38]. Thus, low-dose diuretics were also recommended as second steps.

So far, few randomized trials that compare calcium blockers with low-dose diuretics in type 2 diabetic patients whose hypertension had been inadequately controlled with ARBs have been published. The aim of this study was to compare the efficacy and safety of the calcium blocker azelnidipine with that of the thiazide diuretic trichlormethiazide and the impact of these agents on surrogate markers related to diabetic and hypertensive complications in Japanese type 2 diabetic patients with hypertension who were being treated with the ARB olmesartan.

## Materials and Methods

The protocol for this trial and supporting CONSORT checklist are available as S1 checklist and S3 protocol.

### Participants

The inclusion criteria included men and women between the ages of 20–90 years with adequately controlled diabetes (HbA1c ≤ 7.0%) under lifestyle modification and/or administration of hypoglycemic agents and inadequately controlled hypertension (systolic blood pressure [sBP] ≥ 130 mmHg or diastolic blood pressure [dBP] ≥ 80 mmHg) who were being treated with olmesartan. In this trial, all hypoglycemic agents such as metformin, sulfonylurea, DPP-4 inhibitors, thiazolidinedione, glinide, and α-glucosidase inhibitors were used, whereas insulin and GLP-1 receptor agonist were not. The inclusion criteria had been modified in April 2011 prior to the initiation of this trial. Thus, previous version of the inclusion criteria included patients with impaired glucose tolerance (IGT). Because in many patients diagnosis of IGT was insufficient and there were few patients who met this criteria, subjects were changed to diabetic patients whose HbA1c was ≤ 7.0% under therapy. On the basis of these criteria, 250 patients were screened in the trial.

The following patients were excluded: (i) patients with a history of diabetic ketoacidosis or diabetic coma within 6 months prior to study entry, (ii) patients who had received other antidiabetic agents within 3 months prior to study entry, (iii) patients who underwent a surgical operation during the observation period of this study, (iv) patients with severe infection or severe trauma, (v) patients who were pregnant or lactating, (vi) patients with severe liver dysfunction, (vii) patients with severe renal dysfunction, (viii) patients who had received insulin therapy, (ix) patients who had received steroid therapy, (x) patients with a history of hypersensitivity reaction to azelnidipine or trichlormethiazide, and (xi) patients who were judged as being inappropriate by the physicians in charge.

The Declaration of Helsinki-compliant study protocol was approved by the Ethics Committee of Yokohama City University School of Medicine, Yokohama Seamen's Insurance Hospital, Yokohama Minami Kyosai Hospital, Chigasaki Municipal Hospital, Shonan Fujisawa Tokushukai Hospital, Kanazawa Hospital, Kanagawa Cardiovascuiar and Respiratory Center, St. Joseph’s Hospital and Nagatsuta Kousei General Hospital. All the patients provided their written informed consent.

### Study Protocol

The study was an open-label, randomized controlled trial conducted at 9 institutions in Japan. Recruitment and follow up were performed by physicians according to the criteria mentioned above from August 2011 through to December 2012. Practically, one hundred one patients (azelnidipine group: 50 patients, trichlormethiazide group: 51 patients) were recruited in October 2011, 79 patients (azelnidipine group: 39 patients, trichlormethiazide group: 40 patients) in November 2011, 42 patients (azelnidipine group: 22 patients, trichlormethiazide group: 20 patients) in December 2011, and 18 patients (azelnidipine group: 9 patients, trichlormethiazide group: 9 patients) in January 2012. All the subjects were randomly assigned in a 1:1 ratio to an azelnidipine group (azelnidipine, 16 mg/day) or a trichlormethiazide group (trichlormethiazide, 1 mg/day) using a permuted block method with central computer-based randomization and were followed up for 48 weeks. The range of the olmesartan dose was 10–40 mg/day. The doses of all hypoglycemic agents and hypotensive agents were fixed during this trial. The main outcome measure was the difference in the change in the HbA1c level from the baseline value at 48 weeks between these two groups; key secondary outcomes were the levels of sBP, dBP, fasting plasma glucose (FPG), fasting insulin (FI), inflammation mediators, adiponectin, and markers of lipids, uric acid, liver function and renal function. The registration number for this trial is UMIN 000006081.

### Assessments

At 0, 24, and 48 weeks after randomization, each patient’s body weight and BP level were measured and blood and urine samples were collected to measure the levels of aspartate aminotransaminase (AST), alanine aminotransferase (ALT), gamma-glutamyl transpeptidase (γ-GTP), uric acid (UA), estimated glomerular filtration rate (e-GFR), creatinine (Cr), urine albumin-to-creatinine ratio (UACR), low-density lipoprotein cholesterol (LDL-C), high-density lipoprotein cholesterol (HDL-C), triglyceride (TG), highly sensitive C-reactive protein (hsCRP), adiponectin, FPG, FI, HbA1c, homeostasis model assessment as an index of insulin resistance (HOMA-IR), and the homeostatic model assessment β cell function (HOMA-β). HOMA-IR and HOMA-β represent insulin resistance and pancreatic β-cell function, respectively, and were calculated as follows: HOMA-IR = FI (μU/mL) × FPG (mmol/L)/22.5, and HOMA-β = 20 × FI (μU/mL)/(FPG [mmol/L]- 3.5). For the FPG, FI, HOMA-IR, and HOMA-β assessments, 55 subjects in the azelnidipine group and 57 subjects in the trichlormethiazide group who did not ingest any food or drink, including glucose, before the blood collections at all time points were evaluated.

All the samples from a given individual were labeled using a code and were routinely analyzed by a laboratory at the Showa Medical Science Corporation (Tokyo, Japan). As for the biochemical tests, the HbA1c, glucose, and adiponectin levels were measured using the BioMajesty JCA-BM9030 series (Japan Electron Optics Laboratory, Tokyo, Japan). The FI level was measured using the ADVIA Centaur XP Immunoassay System (Siemens Healthcare Diagnostics Inc., Tokyo, Japan), while the other parameters were measured using the BioMajesty JCA-BM8060 series (Japan Electron Optics Laboratory, Tokyo, Japan). The hsCRP level was measured using a Behring Nephelometer-II (Siemens Healthcare Diagnostics Inc., Tokyo, Japan). The UACR was measured using the BioMajesty JCA-BM2250 series (Japan Electron Optics Laboratory, Tokyo, Japan).

Adverse effects, such as dizziness, palpitation, rash, and edema, were monitored during this trial. Edema was defined as the occurrence or exacerbation of pretibial edema. The occurrence of other symptoms was diagnosed by the physicians in charge.

### Statistical Analysis

The sample size was calculated based on the assumptions that the difference in the 48-week change in the HbA1c level between the azelnidipine group and the trichlormethiazide group would be 0.3% and the standard deviation in the HbA1c level at baseline would be 0.6%. To detect such a significant difference between the two groups under the statistical situation of a power greater than 90% with a two-sided type 1 error rate of 0.05, at least 85 patients were required in each group.

An intention-to-treat analysis was performed for the 240 patients after randomization, and the statistical analysis was conducted using the Statistical Package for the Social Sciences (SPSS -19.0). Missing data from patients who were unable to be followed up or examined for some reason were excluded. The differences in the baseline characteristics between these two groups were analyzed using an unpaired *t*-test, the Mann-Whitney test, or the Fisher exact test, based on the class of variables and the existence of statistical normality in the data distributions. The parameters at 0, 24, and 48 weeks were analyzed in each group using a paired *t*-test or the Wilcoxon signed rank test. The differences in the changes of the parameters at 0–48 weeks were analyzed using an unpaired *t*-test or the Mann-Whitney test, and the differences in the incidence rates of adverse effects were analyzed using the Fisher exact test. A statistically significant difference was defined as a two-sided *P* value < 0.05.

## Results

**Fig 1:**
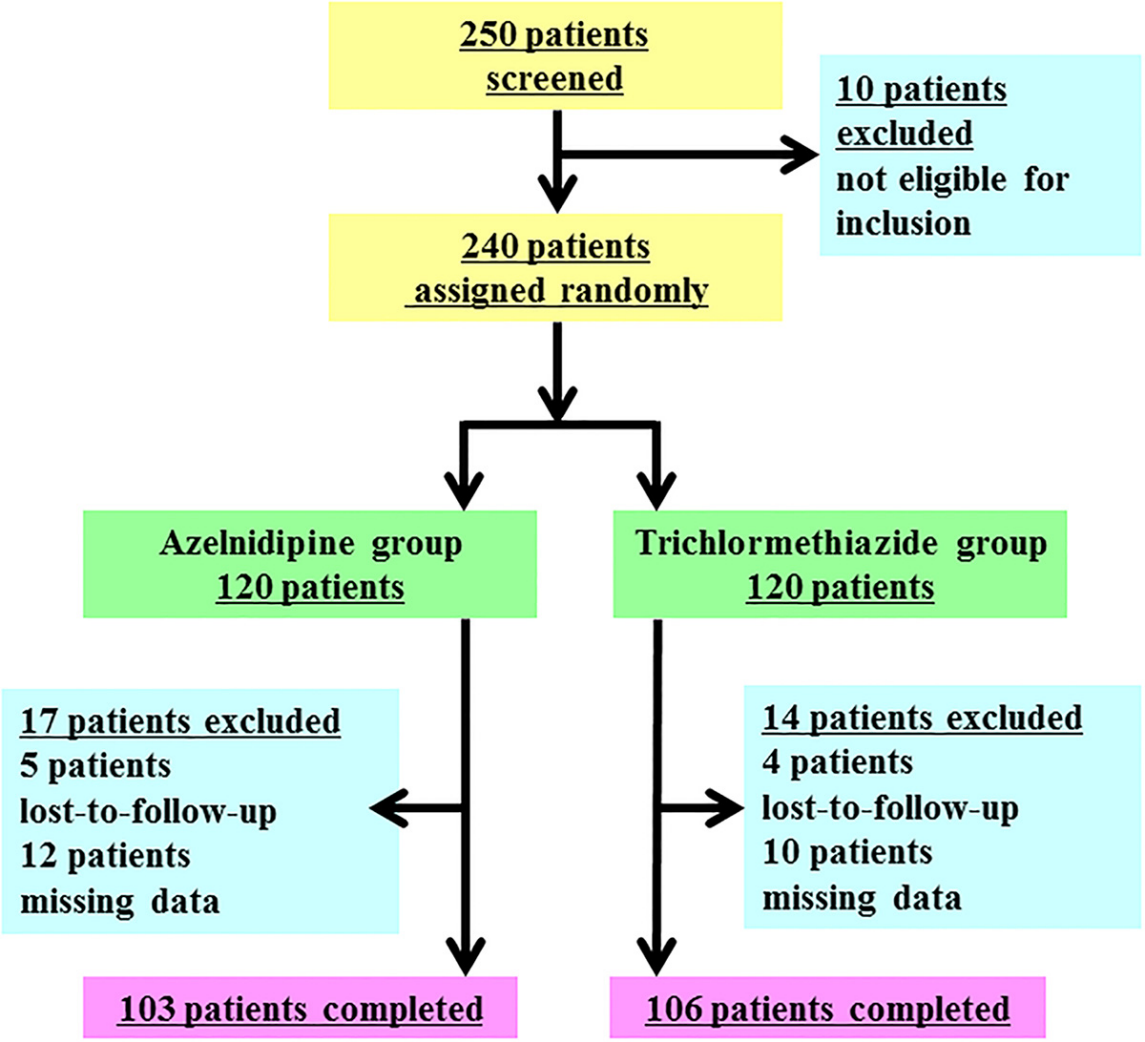
Flow chart of study participants throughout the trial. Of the 250 patients who were enrolled, 209 patients (azelnidipine group: 103 patients, trichlormethiazide group: 106 patients) completed the trial. Seventeen patients in the azelnidipine group and fourteen patients in the trichlormethiazide group were excluded because they were lost-to-follow-up or because of missing data.

Two hundred and fifty patients with adequately controlled diabetes (HbA1c ≤ 7.0%) and inadequately controlled hypertension (systolic blood pressure [sBP] ≥ 130 mmHg or diastolic blood pressure [dBP] ≥ 80 mmHg) with olmesartan were screened for this trial. Ten patients were excluded because they did not meet the inclusion criteria prior to this trial; hence, 240 patients (123 men and 117 women) were randomly assigned to the azelnidipine group or the trichlormethiazide group. Of these 240 patients, 209 patients (azelnidipine group: 103 patients, trichlormethiazide group: 106 patients) completed this trial. Seventeen patients in the azelnidipine group and fourteen patients in the trichlormethiazide group were excluded because they were lost-to-follow-up or because of missing data. Fig 1 shows the flow of the study patients throughout the trial. The final follow-up rate was 87.1%. Table 1 shows the baseline characteristics of the patients and the displayable 14 patients in the excluded 31 patients. No statistically significant differences in the baseline characteristics were observed between these two groups.

**Table 1:** Baseline characteristics of the subjects.

	Azelnidipine group (n = 103)	Trichlormethiazide group (n = 106)
**Age (years)**	**68.4 ± 10.7**	**67.8 ± 11.5**
**Sex (male/female)**	**53 / 50**	**52 / 54**
**Body weight (kg)**	**64.1 ± 11.7**	**63.5 ± 13.9**
**Body mass index (kg/m** ^**2**^ **)**	**25.1 ± 3.7**	**25.0 ± 4.5**
**Systolic blood pressure (mmHg)**	**142 ± 11**	**141 ± 11**
**Diastolic blood pressure (mmHg)**	**80 ± 6**	**79 ± 7**
**HbA1c (%)**	**6.26 ± 0.62**	**6.23 ± 0.60**
**Fasting plasma glucose (mmol/L)**	**6.11 ± 1.17**	**6.00 ± 1.28**
**Fasting insulin (μU/mL)**	**6.72 ± 3.59**	**6.90 ± 4.51**
**Homeostasis model assessment insulin resistance**	**1.85 ± 1.13**	**1.90 ± 1.64**
**Homeostatic model assessment β cell function**	**65.9 ± 57.7**	**64.6 ± 44.0**
**Triglyceride (mmol/L)**	**1.38 ± 0.62**	**1.34 ± 0.65**
**Total cholesterol (mmol/L)**	**4.86 ± 0.80**	**4.86 ± 0.85**
**Low-density lipoprotein cholesterol (mmol/L)**	**2.84 ± 0.67**	**2.84 ± 0.78**
**High-density lipoprotein cholesterol (mmol/L)**	**1.45 ± 0.34**	**1.47 ± 0.36**
**Aspartate aminotransaminase (IU/L)**	**25.4 ± 10.9**	**25.1 ± 12.7**
**Alanine aminotransferase (IU/L)**	**27.5 ± 19.1**	**27.1 ± 20.5**
**Gamma-glutamyl transpeptidase (IU/L)**	**37.7 ± 31.6**	**39.9 ± 37.6**
**Uric acid (μmol/L)**	**331.3 ± 66.6**	**339.6 ± 74.9**
**Estimated glomerular filtration rate (mL/min)**	**71.0 ± 18.7**	**71.8 ± 20.1**
**Creatinine (μmol/L)**	**69.0 ± 22.1**	**67.2 ± 20.3**
**Albumin-to-creatinine ratio (mg/g Cr)**	**106 ± 266**	**96 ± 219**
**Highly sensitive C-reactive protein (mg/mL)**	**0.15 ± 0.34**	**0.11 ± 0.15**
**Adiponectin (ng/mL)**	**3.28 ± 3.52**	**3.18 ± 3.47**
**Baseline characteristics of the displayable 14 patients in 31 patients excluded**	**Azelnidipine group (n = 8)**	**Trichlormethiazide group (n = 6)**
**Age (years)**	**60.6 ± 8.8**	**66.2 ± 15.9**
**Sex (male/female)**	**6 / 2**	**4 / 2**
**Body weight (kg)**	**74.4 ± 15.8**	**70.4 ± 21.0**
**Systolic blood pressure (mmHg)**	**146 ± 16**	**141 ± 7**
**Diastolic blood pressure (mmHg)**	**85 ± 8**	**82 ± 8**
**HbA1c (%)**	**6.08 ± 0.83**	**6.23 ± 0.92**
**The number of patients on oral hypoglycemic agents**	**88**	**93**
**Metformin**	**72 (69.9%)**	**70 (66.0%)**
**Sulfonylurea**	**36 (35.0%)**	**39 (36.8%)**
**DPP-4 inhibitors**	**69 (67.0%)**	**73 (68.9%)**
**Thiazolidinedione**	**7 (6.8%)**	**5 (4.7%)**
**Glinide**	**8 (7.8%)**	**8 (7.5%)**
**α-glucosidase inhibitors**	**7 (6.8%)**	**8 (7.5%)**

The parameters were described as the mean ± SD at baseline in each group.

All the clinical parameters in the azelnidipine and trichlormethiazide groups are shown in Table 2. The differences in the changes in all the parameters from the baseline values to 48 weeks between these two groups are shown in Table 3. At 48 weeks, the mean HbA1c levels had deteriorated in not only the azelnidipine group (from 6.26% ± 0.62% to 6.45% ± 0.79%; *P* < 0.001), but also in the trichlormethiazide group (from 6.23% ± 0.60% to 6.42% ± 0.81%; *P* < 0.001). The respective changes in the HbA1c levels from the baseline values were -0.19% ± 0.52% and -0.19% ± 0.54%, respectively. No significant differences in the changes were observed between the two groups.

**Table 2:** Time courses for clinical parameters in the azelnidipine group and the trichlormethiazide group.

azelnidipine group	0 w	24 w	48 w
**Body weight (kg)**	**64.1 ± 11.7**	**64.2 ± 11.4**	**64.2 ± 11.7**
**Systolic blood pressure (mmHg)**	**142 ± 11**	131 ± 13**	131 ± 13**
**Diastolic blood pressure (mmHg)**	**80 ± 6**	74 ± 7**	73 ± 7**
**HbA1c (%)**	**6.26 ± 0.62**	6.40 ± 0.67**	6.45 ± 0.79**
**Fasting plasma glucose (mmol/L)**	**6.11 ± 1.17**	**6.27 ± 1.28**	**6.27 ± 1.17**
**Fasting insulin (μU/mL)**	**6.72 ± 3.59**	**7.38 ± 4.31**	**7.64 ± 4.35†**
**Homeostasis model assessment insulin resistance**	**1.85 ± 1.13**	**2.11 ± 1.4†**	**2.20 ± 1.60†**
**Homeostatic model assessment β cell function**	**65.9 ± 57.7**	**65.6 ± 57.1**	**60.5 ± 36.5**
**Triglyceride (mmol/L)**	**1.38 ± 0.62**	**1.38 ± 0.69**	**1.56 ± 0.81†**
**Total cholesterol (mmol/L)**	**4.86 ± 0.80**	**4.76 ± 0.80**	**4.81 ± 0.78**
**Low-density lipoprotein cholesterol (mmol/L)**	**2.84 ± 0.67**	**2.79 ± 0.67**	**2.77 ± 0.67**
**High-density lipoprotein cholesterol (mmol/L)**	**1.45 ± 0.34**	**1.45 ± 0.34**	**1.45 ± 0.36**
**Aspartate aminotransaminase (IU/L)**	**25.4 ± 10.9**	**24.5 ± 11.5†**	**26.6 ± 17.1**
**Alanine aminotransferase (IU/L)**	**27.5 ± 19.1**	**26.3 ± 18.6**	**29.0 ± 23.5**
**Gamma-glutamyl transpeptidase (IU/L)**	**37.7 ± 31.6**	**36.9 ± 26.3**	**40.6 ± 32.8††**
**Uric acid (μmol/L)**	**331.3 ± 66.6**	343.8 ± 74.4**	340.2 ± 72.0*
**Estimated glomerular filtration rate (mL/min)**	**71.0 ± 18.7**	67.5 ± 18.9**	68.4 ± 18.9*
**Creatinine (μmol/L)**	**69.0 ± 22.1**	72.5 ± 25.6*	72.5 ± 26.5**
**Albumin-to-creatinine ratio (mg/g Cr)**	**106 ± 266**	**88 ± 218**	**94 ± 219†**
**Highly sensitive C-reactive protein (mg/mL)**	**0.15 ± 0.34**	**0.20 ± 0.41**	**0.15 ± 0.34**
**Adiponectin (ng/mL)**	**3.28 ± 3.52**	**3.10 ± 3.52**	**3.19 ± 3.19**
**trichlormethiazide group**	**0 w**	**24 w**	**48 w**
**Body weight (kg)**	**63.5 ± 13.9**	**63.5 ± 13.9**	**63.6 ± 14.1**
**Systolic blood pressure (mmHg)**	**141 ± 11**	133 ± 12**	134 ± 10**
**Diastolic blood pressure (mmHg)**	**79 ± 7**	75 ± 6**	76 ± 6**
**HbA1c (%)**	**6.23 ± 0.60**	6.44 ± 0.72**	6.42 ± 0.81**
**Fasting plasma glucose (mmol/L)**	**6.00 ± 1.28**	**6.00 ± 1.22**	**6.16 ± 1.55**
**Fasting insulin (μU/mL)**	**6.90 ± 4.51**	**7.57 ± 4.87†**	**8.10 ± 4.60††**
**Homeostasis model assessment insulin resistance**	**1.90 ± 1.64**	**2.05 ± 1.48†**	**2.49 ± 2.30††**
**Homeostatic model assessment β cell function**	**64.6 ± 44.0**	**70.8 ± 53.5**	**69.7 ± 43.9†**
**Triglyceride (mmol/L)**	**1.34 ± 0.65**	**1.60 ± 0.94††**	**1.58 ± 0.89††**
**Total cholesterol (mmol/L)**	**4.86 ± 0.85**	**4.91 ± 0.85**	**4.91 ± 0.91**
**Low-density lipoprotein cholesterol (mmol/L)**	**2.84 ± 0.78**	**2.87 ± 0.83**	**2.87 ± 0.78**
**High-density lipoprotein cholesterol (mmol/L)**	**1.47 ± 0.36**	**1.40 ± 0.36†**	**1.42 ± 0.34†**
**Aspartate aminotransaminase (IU/L)**	**25.1 ± 12.7**	**27.7 ± 31.5**	**24.5 ± 13.0**
**Alanine aminotransferase (IU/L)**	**27.1 ± 20.5**	**29.8 ± 33.5**	**27.2 ± 19.0**
**Gamma-glutamyl transpeptidase (IU/L)**	**39.9 ± 37.6**	**41.0 ± 32.6**	**44.6 ± 44.9†**
**Uric acid (μmol/L)**	**339.6 ± 74.9**	365.8 ± 73.8**	371.8 ± 72.0**
**Estimated glomerular filtration rate (mL/min)**	**71.8 ± 20.1**	67.5 ± 19.9**	68.1 ± 20.9**
**Creatinine (μmol/L)**	**67.2 ± 20.3**	72.5 ±23.0**	71.6 ± 21.2**
**Albumin-to-creatinine ratio (mg/g Cr)**	**96 ± 219**	**64 ± 182††**	**61 ± 180††**
**Highly sensitive C-reactive protein (mg/mL)**	**0.11 ± 0.15**	**0.14 ± 0.28**	**0.29 ± 0.88††**
**Adiponectin (ng/mL)**	**3.18 ± 3.47**	**2.78 ± 2.38†**	**2.94 ± 2.60**

The parameters are described as “mean ± SD” at 0, 24, and 48 weeks. The differences in parameters at 24 and 48 weeks relative to the baseline values (0 weeks) were analyzed using a paired *t*-test (*P < 0.05, **P < 0.01) or a Wilcoxon signed rank test (†*P* < 0.05, ††*P* < 0.01).

**Table 3:** Changes in clinical parameters at 48 weeks relative to baseline values.

	Azelnidipine group (n = 103)	Trichlormethiazide group (n = 106)	*P* value
**Body weight (kg)**	**0.05 (-0.3, 0.4)**	**0.06 (-0.3, 0.4)**	0.945*
**Systolic blood pressure (mmHg)**	**-10.7 (-12.6, -8.8)**	**-7.1 (-8.6, -5.6)**	0.003*
**Diastolic blood pressure (mmHg)**	**-6.6 (-7.9, -5.3)**	**-3.3 (-4.5, -2.2)**	<0.001*
**HbA1c (%)**	**0.19 (0.09, 0.30)**	**0.19 (0.09, 0.30)**	0.988*
**Fasting plasma glucose (mmol/L)**	**0.19 (-0.05, 0.43)**	**0.17 (-0.08, 0.42)**	**0.851†**
**Fasting insulin (μU/mL)**	**0.92 (0.16, 1.68)**	**1.20 (0.41, 1.99)**	**0.444†**
**Homeostasis model assessment insulin resistance**	**0.36 (0.10, 0.63)**	**0.59 (0.09, 1.09)**	**0.571†**
**Homeostatic model assessment β cell function**	**-5.4 ± 42.4**	**5.2 ± 36.2**	**0.205†**
**Triglyceride (mmol/L)**	**0.18 (0.05, 0.31)**	**0.24 (0.12, 0.36)**	**0.325†**
**Total cholesterol (mmol/L)**	**-0.04 (-0.16,0.09)**	**0.05 (-0.07, 0.17)**	0.307*
**Low-density lipoprotein cholesterol (mmol/L)**	**-0.07 (-0.19, 0.04)**	**0.02 (-0.09, 0.12)**	0.080*
**High-density lipoprotein cholesterol (mmol/L)**	**-0.01 (-0.05, 0.03)**	**-0.05 (-0.08, -0.02)**	0.158*
**Aspartate aminotransaminase (IU/L)**	**1.2 (-1.4, 3.8)**	**-0.6 (-2.1, 1.0)**	**0.841†**
**Alanine aminotransferase (IU/L)**	**1.5 (-1.6, 4.5)**	**-0.1 (-2.5, 2.6)**	**0.849†**
**Gamma-glutamyl transpeptidase (IU/L)**	**2.9 (-2.3, 8.2)**	**4.7 (-0.9, 10.2)**	**0.769†**
**Uric acid (μmol/L)**	**8.9 (0.6, 17.2)**	**32.1 (20.2, 43.4)**	0.002*
**Estimated glomerular filtration rate (mL/min)**	**-2.6 (-4.9, -0.3)**	**-3.8 (-5.5, -2.0)**	0.433*
**Creatinine (μmol/L)**	**3.5 (0.9, 5.3)**	**4.4 (1.8, 6.2)**	0.632*
**Albumin-to-creatinine ratio (mg/g Cr)**	**-12.3 (-33.0, 8.4)**	**-34.6 (-52.8, -16.4)**	**0.041†**
**Highly sensitive C-reactive protein (mg/mL)**	**-0.03 (-0.08, 0.08)**	**0.18 (0.01, 0.35)**	**0.017†**
**Adiponectin (ng/mL)**	**-0.05 (-0.30, 0.19)**	**-0.24 (-0.66, 0.18)**	**0.529†**

The changes in the parameters between 0 and 48 weeks were described as the mean (95% confidence interval) and were analyzed using an unpaired *t*-test* or Mann-Whitney test†.

At 48 weeks, the mean sBP/dBP levels in the azelnidipine and trichlormethiazide groups improved from 142 ± 11/80 ± 6 mmHg to 131 ± 13/73 ± 7 mmHg (*P* < 0.001 for both sBP and dBP) and from 141 ± 11/79 ± 7 mmHg to 134 ± 10/76 ± 6 mmHg mmHg (*P* < 0.001 for both sBP and dBP), respectively. The respective changes in sBP and dBP between the two groups (azelnidipine vs trichlormethiazide) were -10.7 ± 9.6 vs -7.1 ± 7.7 mmHg (*P* = 0.003) and -6.6 ± 6.6 vs -3.3 ± 6.1 mmHg (*P* < 0.001), respectively.

The mean BW and FPG in the azelnidipine and trichlormethiazide groups had not changed significantly at 48 weeks, but the mean FI and HOMA-IR had deteriorated from 6.72 ± 3.59 μU/mL to 7.64 ± 4.35 μU/mL (*P* = 0.031) and from 1.85 ± 1.13 to 2.20 ± 1.60 (*P* = 0.021) in the azelnidipine group and from 6.90 ± 4.51 μU/mL to 8.10 ± 4.60 μU/mL (*P* = 0.002) and from 1.90 ± 1.64 to 2.49 ± 2.30 (*P* = 0.001) in the trichlormethiazide group, respectively. There was no significant deference in the two groups regarding the deterioration of FI and HOMA-IR. The mean TG levels in both groups had deteriorated significantly (*P* = 0.033 and *P* = 0.001, respectively), but the mean TC, LDL-C, and HDL-C levels had not deteriorated.

At 48 weeks, the mean eGFR, Cr levels, and UA levels were slightly exacerbated in the azelnidipine group from 71.0 ± 18.7 IU/L to 68.4 ± 18.9 IU/L (*P* = 0.018), from 69.0 ± 22.1 μmol/L to 72.5 ± 26.5 μmol/L (*P* = 0.002), and from 331.3 ± 66.6 μmol/L to 340.2 ± 72.0 μmol/L (*P* = 0.044), respectively, and in the trichlormethiazide group from 71.8 ± 20.1 IU/L to 68.1 ± 20.9 IU/L (*P* < 0.001), from 67.2 ± 20.3 μmol/L to 71.6 ± 21.2 μmol/L (*P* < 0.001), and from 339.6 ± 74.9 μmol/L to 371.8 ± 72.0 μmol/L (*P* < 0.001), respectively. However, no significant differences in the changes from the baseline values were observed between the two groups except for the UA levels at 48 weeks (8.9 ± 44.0 μmol/L in the azelnidipine group and 32.1 ± 58.9 μmol/L in the trichlormethiazide group; *P* = 0.002). The UACR improved in the azelnidipine group from 106 ± 266 mg/g Cr to 94 ± 219 mg/g Cr (*P* = 0.045) and in the trichlormethiazide group from 96 ± 219 mg/g Cr to 61 ± 180 mg/g Cr (*P* = 0.002) at 48 weeks. The changes in the UACR from the baseline values were -12.3 ± 105.8 mg/g Cr and -34.6 ± 94.4 mg/g Cr (*P* = 0.041), respectively.

In the azelnidipine and trichlormethiazide groups, dizziness (12 patients [11.7%] and 16 patients, [15.1%], respectively), edema (16 patients [15.5%] and 7 patients [6.6%], respectively; *P* = 0.047), and palpitation (0 patients [0%] and 2 patients [1.9%], respectively) were observed during the 48 weeks. No severe cases of adverse effects were observed in either group during the trial.

## Discussion

In this trial, we compared the calcium blocker azelnidipine with the thiazide diuretic trichlormethiazide with respect to their efficacy and adverse events in patients with adequately controlled diabetes and inadequately controlled hypertension who were being treated with the angiotensin receptor blocker olmesartan. We attempted to evaluate which agent is preferable as an additional antihypertensive for patients who have been treated with angiotensin receptor blockers. Our results were analyzed in intention-to-treat analyses, on the other hand, they were equal to the results in per-protocol analyses as described in Table 4. This trial was designed to imitate one of the most frequent daily clinical situations encountered by not only diabetologists, but also general practitioners who have to control diabetes and hypertension even though they are not specialists.

**Table 4:** Changes in clinical parameters at 48 weeks relative to baseline values in per-protocol analyses.

	Azelnidipine group (n = 99)	Trichlormethiazide group (n = 102)	*P* value
**Systolic blood pressure (mmHg)**	**-10.6 (-12.5, -8.7)**	**-7.1 (-8.6, -5.6)**	0.006*
**Diastolic blood pressure (mmHg)**	**-6.6 (-7.9, -5.3)**	**-3.3 (-4.6, -2.2)**	<0.001*
**HbA1c (%)**	**0.18 (0.09, 0.27)**	**0.19 (0.09, 0.32)**	0.901*
**Albumin-to-creatinine ratio (mg/g Cr)**	**-10.1 (-31.0, 10.7)**	**-35.8 (-54.5, -17.2)**	**0.035†**

The changes in the parameters between 0 and 48 weeks were described as the mean (95% confidence interval) and were analyzed using an unpaired *t*-test* or Mann-Whitney test†.

Of note, azelnidipine and trichlormethiazide similarly exacerbated glycemic control in type 2 diabetic patients with hypertension. At 48 weeks, the HbA1c levels, FI, and HOMA-IR were significantly exacerbated in both groups. These results indicate that the exacerbation of glucose metabolism is caused by the elevation of insulin resistance.

In American and European trials, a combination of the ACE inhibitor delapril and the calcium blocker manidipine did not significantly change the HbA1c levels [39–40]; on the other hand, a combination of the ACE inhibitor trandolapril and the calcium blocker verapamil significantly exacerbated the HbA1c levels [41]. The differences between these trials may be explained by differences in race, treatment agents, and study designs. However, the effect of calcium blockers on glucose metabolism remains controversial, and further detailed investigation is needed.

On the other hand, our results indicate that the impact on glucose metabolism of fixed low-dose trichlormethiazide is equivalent to that of the calcium blocker azelnidipine. However, the change in HbA1c level was unexpectedly small, despite the fact that diuretics reportedly increase the incidence of adverse events in a dose-dependent manner [36]. Even though most of the previous trials have shown diuretics to exacerbate glucose metabolism [20, 33, 39], low-dose diuretics are reportedly tolerable [38] and the fixed low-dose thiazide diuretics are likely to be more tolerable in Japanese type 2 diabetes patients with regard to glucose metabolism than previously expected.

In spite of the fact that azelnidipine was more effective for BP control than trichlormethiazide, trichlormethiazide was more effective for reducing the UACR than azelnidipine in type 2 diabetic patients with hypertension. This result is consistent with a previous report [32]. However, the reasons for the greater reduction in the UACR in the trichlormethiazide group cannot be explained from our results. In addition, there were few studies which investigated the amplitudes of hypotensive effects of azelnidipine and fixed low-dose diuretics. Further investigation and comparison with our results is needed.

In the azelnidipine group, dizziness and edema were respectively observed in 12 patients (11.7%) and 16 patients (15.5%) in this trial. The incidence rates of these adverse effects were higher than in another domestic study using calcium blockers [22]. In American and European patients, the reported incidences of dizziness and edema induced by calcium blockers were 9.8%-20.7% [31–32] and 19.0%-31.2% [21, 31–32], compared with 2.2% and 1.5% [22] in Japanese patients. This result may be due to differences in race, the dose of the calcium blockers, or the method used to evaluate these adverse effects.

The present study had some limitations. First, this trial did not have a placebo group. Therefore, we cannot exclude the possibility that the exacerbation of the parameters during the 48 weeks was due to the progression of diabetic and hypertensive complications. Second, some patients failed to observe the instructions for collecting blood under fasting conditions. This limitation likely affected the FPG, FI, HOMA-IR, and HOMA-β parameters, as well as others. Third, the doses of ARBs and the calcium blockers used in the Japanese trials differed from those used in American and European trials. Fourth, in this trial, HbA1c levels of all participants were ≤ 7.0% because we were afraid of worsening glycemic control especially in trichlormethiazide group. But our results suggested that fixed low-dose thiazide diuretics are likely to be as tolerable as calcium blockers for glucose metabolism. Further investigation of similar effects in patients with HbA1c ≥ 7.0% is desirable. Fifth, in this trial, most recruitment was performed from October 2011 through to January 2012, even though blood pressure is generally affected by seasonal variations. Therefore, we cannot deny the fact that our results were likely to be affected by seasonal variations.

The ADA recommends that people with diabetes and hypertension should be treated so as to achieve a sBP/dBP goal of 140/80 mmHg [2], since extreme BP control does not reduce the risk for cardiovascular disease in type 2 diabetic patients with hypertension [35, 42]. On the other hand, the sBP/dBP goal of the JSH is 130/80 mmHg [5]. Of note, the incidence of cardiovascular disease is lower and the incidence of stroke is higher in Japanese patients than in American and European patients.

In conclusion, azelnidipine was more effective for controlling blood pressure than trichlormethiazide, whereas trichlormethiazide was more effective for reducing albuminuria than azelnidipine. Both of these agents, however, similarly exacerbated glycemic control in type 2 diabetic patients with hypertension.

## Supporting Information

S1 checklist CONSORT checklist of this trial. (DOC)

S1 protocol This protocol was modified in 2011. (DOC)

S2 protocol This protocol was used in this trial. (DOC)

S3 protocol English version of this protocol. (DOC)

S4 protocol Main points of modification from the 2010 protocol to the 2011 protocol. (DOC)

S1 IRB The list of institutional review board members in Yokohama City University Hospital in 2011. (XLS)
