# Supplementary material for: Comparison of Azelnidipine and Trichlormethiazide in Japanese Type 2 Diabetic Patients with Hypertension: The COAT Randomized Controlled Trial
Source: PLoS One. 2015 May 4;10(5):e0125519. doi: 10.1371/journal.pone.0125519 (PMC4418830; doi:10.1371/journal.pone.0125519)
Supplement: S2 protocol — (DOC) [file pone.0125519.s003.doc]

様式第２号

平成23年9月12日

Sep 12, 2011

臨床研究実施概要書

Study protocol for our clinical research

【研究責任者】内分泌・糖尿病内科　大学院3年　瀧端　正博

Representative: Department of Endocrinology and Metabolism

Third year graduate student; Masahiro Takihata

| １　開発・研究名  Title | 「オルメサルタンとの併用療法におけるアゼルニジピン及びトリクロルメチアジドの耐糖能への影響の比較: 耐糖能障害合併高血圧患者における多施設共同無作為化比較試験: COAT試験」  「Comparison of azelnidipine and trichlormethiazide in Japanese type 2 diabetic patients with hypertension who are being treated with the olmesartan: the COAT randomized controlled trial」 |
| --- | --- |
| ２　研究の背景  （この研究がなされるに至った過程や結果として予想される効果）  Background | 境界型糖尿病、糖尿病における血圧管理は動脈硬化性合併症の予防の観点から極めて重要である。血圧管理目標を達成するために、RAS系降圧薬に加えカルシウム拮抗薬や利尿薬を併用することは、臨床においてはしばしば遭遇する場面であり、現在の高血圧治療ガイドラインにおいては、カルシウム拮抗薬と少量利尿薬は第2選択薬として同等の位置づけにある。  It is important to control blood pressure in diabetic patients for prevention of arteriosclerosis. We often use RAS inhibitors as the first step, but the additional use of antihypertensive medications is controversial. In the guideline of Japan Hypertension Society in 2009, both calcium blockers and diuretics are recommended as the second step.  これら薬剤の耐糖能に対する影響については以前より多くの基礎および臨床研究において検討されている。カルシウム拮抗薬についてはインスリン抵抗性改善作用や抗炎症作用が空腹時血糖を改善させ、ARBとの併用で効果が増強されるという報告がある。カルシウム拮抗薬の比較では、アゼルニジピンの骨格筋へのグルコース取り込み増強効果が高いとの報告がある。  The impacts of these agents on glucose tolerance were investigated by many basic studies and clinical studies. Some reports showed that calcium blockers improved insulin resistance and fasting plasma glucose with angiotensin receptor blockers (ARBs). Especially, it is reported that azelnidipine is more effective for improvement of glucose metabolism than other calcium blockers because azelnidipine enhances glucose transport to skeletal muscle.  一方で利尿薬はインスリン感受性を低下させ、また利尿薬による血清K値の低下が血糖値上昇に関連しているという報告があるが、利尿薬とARBの併用により耐糖能の増悪は相殺されるという報告もある。また、カルシウム拮抗薬や利尿薬の耐糖能や大血管障害に対する検討は多くの臨床研究での報告があるが、細小血管障害についてはその数は少数である。  Whereas diuretics decrease insulin sensitivity by means of lowering of serum K level, some reports showed that the problem might be resolved by ARBs. In addition, there were many studies of the impacts on glucose tolerance and diabetic macroangiopathy, but were few studies of diabetic microangiopathy.  そこで今回は、降圧薬併用療法における、その有用性、安全性を検証する目的で、高血圧症を有する境界型糖尿病患者および糖尿病患者を対象に、オルメサルタンとアゼルニジピン、またはオルメサルタンと少量のサイアザイド系利尿薬を12ヶ月間投与し、これら薬剤の糖代謝への影響及び忍溶性と耐糖能異常に関連した腎機能障害への影響について比較検討する。  　To investigate the efficacy and safety of the calcium blockers and the diuretics with ARBs and the impact of these agents on surrogate markers related to diabetic and hypertensive complications, we compare azelnidipine with trichlormethiazide in Japanese type 2 diabetic patients with hypertension who were being treated with the olmesartan during 12 months. |
| ３　目　的  Aims | 高血圧症合併の2型糖尿病および境界型糖尿病患者におけるオルメサルタンとアゼルニジピンまたはオルメサルタンと少量サイアザイド系利尿薬併用による糖代謝に与える影響と耐糖能異常に関連した腎機能障害への影響を比較する。  　The aim of this study is to compare the efficacy and safety of the calcium blocker azelnidipine with that of the thiazide diuretic trichlormethiazide and the impact of these agents on surrogate markers related to diabetic and hypertensive complications in Japanese type 2 diabetic patients with hypertension who are being treated with the ARB olmesartan. |
| ４　意義・独創性　等  Significance  and originality | 境界型糖尿病および糖尿病患者における血圧管理は動脈硬化性合併症の予防の観点から極めて重要である。ARBに加えて降圧薬併用療法を行う際のカルシウム拮抗薬または少量利尿薬の選択には、耐糖能への影響、細小血管障害進展への影響についてさらに検討を重ねることが、より安全性の高い治療法の確立へつながり、それには大規模な前向きの比較試験が必要である。今回は１群85例でありこれまでの比較研究と比べて同等以上の規模であることより質の高い研究と考える。  It is important to control blood pressure in diabetic patients for prevention from arteriosclerosis and to investigate the impact of calcium blockers and thiazide diuretics on surrogate markers related to diabetic and hypertensive complications. Our study is more appropriate than other studies because there are no less than 85 patients in each group. |
| ５　対象と方法  Subjects and methods | 1. 対象（年齢を含む）   Subjects (including age)  横浜市立大学附属病院並びに当科関連病院に通院中の2型糖尿病患者または境界型糖尿病を有する高血圧症患者  症例数：240例  年齢：20歳以上90歳以下  Patients with adequately controlled diabetes under lifestyle modification and/or administration of hypoglycemic agents and inadequately controlled hypertension in Yokohama City University hospital or affiliated hospital.  Target sample size: 240 patients  Age: 20-90 years old  以下の患者は対象外とする  1) 過去6カ月以内に重症ケトーシス、糖尿病性昏睡または前昏睡の既往のある患者  2) 経口糖尿病薬を使用中の場合、組み入れ前3か月間に経口糖尿病薬を変更した患者  3) 観察期間中に手術を施行された患者  4) 重症感染症、重篤な外傷のある患者  5) 妊娠または妊娠している可能性のある婦人および授乳中の患者  6) 重篤な肝機能障害のある患者  7) 重篤な腎機能障害のある患者  8) インスリン使用中の患者  9) ステロイド使用中の患者  10) アゼルニジピンまたはトリクロルメチアジドに対し過敏症の既往歴のある患者  11) その他、試験担当医師が本試験に不適切と判断した症例  Key exclusion criteria  1. Patients with history of diabetic ketoacidosis, or diabetic coma within 3 months prior to the study entry.  2. Patients who had received other antidiabetic agents within 3 months prior to study entry.  3. Patients who received surgical operation during the observation period of this study.  4. Patients with severe infection or severe trauma.  5. Patients with during pregnancy or lactation.  6. Patients with severe liver dysfunction.  7. Patients with severe renal dysfunction.  8. Patients who received insulin therapy.  9. Patients who received steroid therapy.  10. Patients with history of hypersensitivity reaction to azelnidipine or trichlormethiazide.  11. Patients determined to be inappropriate by physician.   1. 具体的実施方法   2型糖尿病または境界型糖尿病で高血圧治療ガイドライン2009の血圧管理目標値を未達成の患者に対し、インフォームドコンセントを得た後、オルメサルタン5～40mgを開始する。オルメサルタン内服開始後も血圧管理目標未達成である場合に試験組み入れとなり、無作為に割り付けした試験薬併用を開始する。血圧管理目標は130/80ｍｍHg未満とする。治療内容としては、オルメサルタン5～40mgを基礎薬とし、アゼルニジピン16mgまたはトリクロロメチアジド1mgを追加する。  Methods  Patients who are being treated with olmesartan with adequately controlled diabetes under lifestyle modification and/or administration of hypoglycemic agents and inadequately controlled hypertension (the criteria was under BP 130/80 based on guideline of Japan Hypertension Society in 2009) are enrolled. Participants are randomly assigned to an azelnidipine group or a trichlormethiazide group. The dose of olmesartan, azelnidipine and trichlormethiazide are 5-40 mg/day, 16 mg/day and 1 mg/day, respectively.  【観察および検査項目】  割り付け時（降圧薬併用療法開始時）、併用療法開始24週後、48週後の空腹時採血、採尿により糖代謝及び以下の項目について評価する。   1. 糖代謝：空腹時血糖、HbA1c、IRI、HOMA-IR 2. 糖代謝関連項目：高分子アディポネクチン 3. 抗炎症マーカー：hsCRP 4. 耐糖能異常合併症：尿中アルブミン/クレアチニン比   Primary outcomes and key secondary outcomes.  At 0, 24, and 48 weeks after randomization, each patient’s body weight and BP level are measured and blood and urine samples are collected.   1. Glucose metabolism: fasting plasma glucose, HbA1c, IRI, HOMA-IR 2. Related marker of glucose metabolism: high molecular adiponectin 3. Marker of inflammation: highly sensitive C-reactive protein 4. Diabetic and hypertensive complications: Urine albumin-to-creatinine ratio |
| ６　予想される結果  （研究から想定される成果など）  Expected results  (products from this study) | ARBとの併用で、アゼルニジピンは、耐糖能、耐糖能異常に関連した腎機能障害に影響を与えることなく血圧管理目標を達成できる。  Azelnidipine improve blood pressure level under the criteria of Japan Hypertension Society and glucose intolerance without renal dysfunction in Japanese type 2 diabetic patients with hypertension who are being treated with the ARB olmesartan. |
| ７　研究期間  Study period | 平成22年10月 1日　～　平成23年12月31日  From October 1, 2010 through to December 31, 2012. |
| ８　対象症例数  Target sample size | 240症例  240 patients |
| ９　研究にかかる費用  （研究費等の明確化）  Cost of this research  (Conflicts of Interest) | ・■奨学寄附金　・受託研究費・■教育研究費　・その他（　　　）  ・■Research grant from companies ・Contract research fund ・■Education research fund by the institute　・others ( ) |
| 10　その他の特記事項  　Special Notes | 1. 安全性の確保   治療的研究である本研究において、個人の名前・情報が第三者に漏洩することは無い。また、患者の血液検体を用いての血中濃度測定もほとんどが保険診療の範囲内であり，病院血液検査室において施行され，患者からの組織・細胞採取やゲノムDNA抽出は一切行わない。よって個人の遺伝子関連の情報にかかわる研究内容ではない。本研究の学会発表・論文報告においても個人を特定できる情報は一切公表しないものである。  Reserve for safety  　We prohibited leakage of private names and information in our study. Extraction of genomic DNA, cells and tissue from our participants was prohibited. All tests were performed at laboratory in each hospital and covered by health care services provided by health insurance in Japan. We must not publish any information about individual in presentation.   1. 副作用等に対する配慮   本研究において使用される薬剤はすでに保険診療上認められており，科学的かつ慎重に行われる。もしも本研究の期間中あるいは終了後に対象者において副作用などの健康障害が生じた場合には，医師が適切な診療と治療を行う。有害事象が発生した場合は適切な診療と処置を行い、関係製薬会社に有害事象発生を連絡する。有害事象には各種検査値以上も含める。有害事象名、発現日、程度、重篤度、転帰、試験薬との因果関係をカルテ及び症例報告書に記載する。尚、本件急は保険適応内での使用範囲に留まっているため、特別な補償は行わない。通常の診療を受けた際に発生した健康被害や医療事故と全く同じ扱いになる。  Consideration for adverse effects  Administration of olmesartan, azelnidipine and trichlormethiazide is covered by health care services provided by health insurance, and these agents are administrated carefully. If unexpected adverse effects happened, all participants should be cared by physicians and the fact should be disclosed and recorded on each medical chart. In our study, there is no particular　compensation for adverse events because all treatments are covered by health care services provided by health insurance. It seems that this compensation is equal to medical accidents caused by usual treatment.   1. 将来の疾病予防・新しい治療方法　等への貢献   2型糖尿病患者および境界型糖尿病患者における血圧管理で、オルメサルタンとの併用の場合、安全かつ有効な薬剤治療としてアゼルニジピンまたはサイアザイド系利尿薬のいずれが最も適しているのか明らかになると考えられる。  Contribution to prevention of disease and development of new methods for therapy.  It will be uncovered which agent is preferable to blood pressure control in type 2 diabetic patients with hypertension who are being treated with the olmesartan. |

＊先進医療を申請する場合は、その実施計画書を添付すること。
