# Supplementary material for: Comparison of Azelnidipine and Trichlormethiazide in Japanese Type 2 Diabetic Patients with Hypertension: The COAT Randomized Controlled Trial
Source: PLoS One. 2015 May 4;10(5):e0125519. doi: 10.1371/journal.pone.0125519 (PMC4418830; doi:10.1371/journal.pone.0125519)
Supplement: S3 protocol — (DOC) [file pone.0125519.s004.doc]

File style 2

Sep 12, 2011

Study protocol for our clinical research

Representative: Department of Endocrinology and Metabolism

Third year graduate student; Masahiro Takihata

| １　Title | 「Comparison of azelnidipine and trichlormethiazide in Japanese type 2 diabetic patients with hypertension who are being treated with the olmesartan: the COAT randomized controlled trial」 |
| --- | --- |
| ２　Background | It is important to control blood pressure in diabetic patients for prevention of arteriosclerosis. We often use RAS inhibitors as the first step, but the additional use of antihypertensive medications is controversial. In the guideline of Japan Hypertension Society in 2009, both calcium blockers and diuretics are recommended as the second step.  The impacts of these agents on glucose tolerance were investigated by many basic studies and clinical studies. Some reports showed that calcium blockers improved insulin resistance and fasting plasma glucose with angiotensin receptor blockers (ARBs). Especially, it is reported that azelnidipine is more effective for improvement of glucose metabolism than other calcium blockers because azelnidipine enhances glucose transport to skeletal muscle.  Whereas diuretics decrease insulin sensitivity by means of lowering of serum K level, some reports showed that the problem might be resolved by ARBs. In addition, there were many studies of the impacts on glucose tolerance and diabetic macroangiopathy, but were few studies of diabetic microangiopathy.  　To investigate the efficacy and safety of the calcium blockers and the diuretics with ARBs and the impact of these agents on surrogate markers related to diabetic and hypertensive complications, we compare azelnidipine with trichlormethiazide in Japanese type 2 diabetic patients with hypertension who were being treated with the olmesartan during 12 months. |
| ３　Aims | The aim of this study is to compare the efficacy and safety of the calcium blocker azelnidipine with that of the thiazide diuretic trichlormethiazide and the impact of these agents on surrogate markers related to diabetic and hypertensive complications in Japanese type 2 diabetic patients with hypertension who are being treated with the ARB olmesartan. |
| ４　Significance  and originality | It is important to control blood pressure in diabetic patients for prevention from arteriosclerosis and to investigate the impact of calcium blockers and thiazide diuretics on surrogate markers related to diabetic and hypertensive complications. Our study is more appropriate than other studies because there are no less than 85 patients in each group. |
| ５　Subjects and methods | 1. Subjects (including age)   Patients with adequately controlled diabetes under lifestyle modification and/or administration of hypoglycemic agents and inadequately controlled hypertension in Yokohama City University hospital or affiliated hospital.  Target sample size: 240 patients  Age: 20-90 years old  Key exclusion criteria  1. Patients with history of diabetic ketoacidosis, or diabetic coma within 3 months prior to the study entry.  2. Patients who had received other antidiabetic agents within 3 months prior to study entry.  3. Patients who received surgical operation during the observation period of this study.  4. Patients with severe infection or severe trauma.  5. Patients with during pregnancy or lactation.  6. Patients with severe liver dysfunction.  7. Patients with severe renal dysfunction.  8. Patients who received insulin therapy.  9. Patients who received steroid therapy.  10. Patients with history of hypersensitivity reaction to azelnidipine or trichlormethiazide.  11. Patients determined to be inappropriate by physician.  Methods  Patients who are being treated with olmesartan with adequately controlled diabetes under lifestyle modification and/or administration of hypoglycemic agents and inadequately controlled hypertension (the criteria was under BP 130/80 based on guideline of Japan Hypertension Society in 2009) are enrolled. Participants are randomly assigned to an azelnidipine group or a trichlormethiazide group. The dose of olmesartan, azelnidipine and trichlormethiazide are 5-40 mg/day, 16 mg/day and 1 mg/day, respectively.  Primary outcomes and key secondary outcomes.  At 0, 24, and 48 weeks after randomization, each patient’s body weight and BP level are measured and blood and urine samples are collected.   1. Glucose metabolism: fasting plasma glucose, HbA1c, IRI, HOMA-IR 2. Related marker of glucose metabolism: high molecular adiponectin 3. Marker of inflammation: highly sensitive C-reactive protein 4. Diabetic and hypertensive complications: Urine albumin-to-creatinine ratio |
| ６　Expected results  (products from this study) | Azelnidipine improve blood pressure level under the criteria of Japan Hypertension Society and glucose intolerance without renal dysfunction in Japanese type 2 diabetic patients with hypertension who are being treated with the ARB olmesartan. |
| ７　Study period | From October 1, 2010 through to December 31, 2012. |
| ８　Target sample size | 240 patients |
| ９　Cost of this research  (Conflicts of Interest) | ・■Research grant from companies ・Contract research fund ・■Education research fund by the institute　・others ( ) |
| 10　Special Notes | (1) Reserve for safety  　We prohibited leakage of private names and information in our study. Extraction of genomic DNA, cells and tissue from our participants was prohibited. All tests were performed at laboratory in each hospital and covered by health care services provided by health insurance in Japan. We must not publish any information about individual in presentation.  (2) Consideration for adverse effects  Administration of olmesartan, azelnidipine and trichlormethiazide is covered by health care services provided by health insurance, and these agents are administrated carefully. If unexpected adverse effects happened, all participants should be cared by physicians and the fact should be disclosed and recorded on each medical chart. In our study, there is no particular　compensation for adverse events because all treatments are covered by health care services provided by health insurance. It seems that this compensation is equal to medical accidents caused by usual treatment.  (3) Contribution to prevention of disease and development of new methods for therapy.  It will be uncovered which agent is preferable to blood pressure control in type 2 diabetic patients with hypertension who are being treated with the olmesartan. |
