# Supplementary material for: Comparison of Azelnidipine and Trichlormethiazide in Japanese Type 2 Diabetic Patients with Hypertension: The COAT Randomized Controlled Trial
Source: PLoS One. 2015 May 4;10(5):e0125519. doi: 10.1371/journal.pone.0125519 (PMC4418830; doi:10.1371/journal.pone.0125519)
Supplement: S4 protocol — (DOC) [file pone.0125519.s005.doc]

様式第９号

平成23年8月11日

Aug 11, 2011

臨床研究計画変更届出書

Modified study protocol for our clinical research

横浜市立大学附属病院長

The director of　Yokohama City University Hospital

研究責任者

所属　横浜市立大学附属病院　内分泌・糖尿病内科　大学院3年

　　職・氏名　　　　　　　　 瀧端　正博　　　　㊞

　　　　　　　　　　　　　　 総括責任者

　　　　　　　　　　　　　　　 所属　横浜市立大学

職・氏名　　　　　　　　　　　　　　 寺内　康夫 ㊞

Representative: Yokohama City University Hospital

Department of Endocrinology and Metabolism

Third year graduate student; Masahiro Takihata

Director: Yokohama City University Hospital

Department of Endocrinology and Metabolism

Professor; Yasuo Terauchi

次の開発・研究について、研究実施計画書を変更したいので、届け出ます。

We submit the modified study protocol for our clinical research.

【開発・研究名：オルメサルタンとの併用療法におけるアゼルニジピンおよびサイアザイド系利尿薬の耐糖能への影響の比較：耐糖能異常合併高血圧患者における多施設共同無作為化比較試験】

【承認番号　B100513010】

【変更（予定）年月日：平成23年9月12日】

【Title: Comparison of azelnidipine and trichlormethiazide in Japanese type 2 diabetic patients with hypertension who were being treated with the olmesartan】

【Authorization number: B100513010】

【Prearranged date: Sep 12, 2011】

| 変更点  modification | 変更前  before the change | 変更後  after the change |
| --- | --- | --- |
| 研究責任者  Representative | 横浜市立大学附属病院  内分泌・糖尿病内科  教授　寺内　康夫  Yokohama City University Hospital  Department of Endocrinology and Metabolism  Professor; Yasuo Terauchi | 横浜市立大学附属病院  内分泌・糖尿病内科  大学院3年　瀧端　正博  Yokohama City University Hospital Department of Endocrinology and Metabolism  Third year graduate student; Masahiro Takihata |
| 対象患者  subject | 1) 組み入れ前3か月間に入院歴のある患者  2) 組み入れ前3か月間の体重変化＞1kgの患者  3) 経口糖尿病薬を使用中の場合、組み入れ前6か月間に経口糖尿病薬を変更した患者  4) インスリン治療を受けている患者  5) 抗GAD抗体陽性などの1型糖尿病の患者  6) 2次性高血圧の疑いのある患者  7) 増殖網膜症以上の糖尿病性網膜症を認める患者  8) 拡張期血圧≧120ｍｍHgの者  9) 過去または現在において心不全を有する者  10) 重篤な肝疾患、または腎疾患を有する患者  11) 悪性疾患を有する患者  12) 妊娠中または妊娠の可能性がある者  13) その他医師が不適当と認めた者  Key exclusion criteria   1. Patients with history of hospitalization within 3 months prior to the study entry. 2. Patients whose body weight was changed over 1kg. 3. Patients who had received other antidiabetic agents within 6 months prior to study entry. 4. Patients who received insulin therapy. 5. Patients who was diagnosed with type 1 diabetes by Anti-GAD Antibody. 6. Patients who had secondary hypertension. 7. Patients who had severe retinopathy. 8. Patients whose diastolic blood pressure was over 120mmHg. 9. Patients with severe cardiac failure. 10. Patients with severe liver dysfunction or renal dysfunction. 11. Patients with malignant tumor. 12. Patients with during pregnancy or lactation. 13. Patients determined to be inappropriate by physician. | 1) 過去6カ月以内に重症ケトーシス、糖尿病性昏睡または前昏睡の既往のある患者  2) 経口糖尿病薬を使用中の場合、組み入れ前3か月間に経口糖尿病薬を変更した患者  3) 観察期間中に手術を施行された患者  4) 重症感染症、重篤な外傷のある患者  5) 妊娠または妊娠している可能性のある婦人および授乳中の患者  6) 重篤な肝機能障害のある患者  7) 重篤な腎機能障害のある患者  8) インスリン使用中の患者  9) ステロイド使用中の患者  10) アゼルニジピンまたはトリクロルメチアジドに対し過敏症の既往歴のある患者  11) その他、試験担当医師が本試験に不適切と判断した症例  Key exclusion criteria   1. Patients with history of diabetic ketoacidosis, or diabetic coma within 3 months prior to the study entry. 2. Patients who had received other antidiabetic agents within 3 months prior to study entry. 3. Patients who received surgical operation during the observation period of this study. 4. Patients with severe infection or severe trauma. 5. Patients with during pregnancy or lactation. 6. Patients with severe liver dysfunction. 7. Patients with severe renal dysfunction. 8. Patients who received insulin therapy. 9. Patients who received steroid therapy. 10. Patients with history of hypersensitivity reaction to azelnidipine or trichlormethiazide. 11. Patients determined to be inappropriate by physician. |
| 研究期間  Study period | 平成22年 6月 1日　～　平成24年 6月 1日  From June 1, 2010 through to June 1, 2012. | 平成23年10月 1日　～　平成24年12月31日  From October 1, 2011 through to December 31, 2012. |
| 患者年齢  Age of subjects | 20-80歳  Age: 20-80 years old | 20-90歳  Age: 20-90 years old |
| 目標患者数  Target sample size | 140症例  140 patients | 240症例  240 patients |
| 採血ポイントの  変更  The change of schedule in blood collection | 0週、12週、48週  0w, 12w, 48w | 0週、24週、48週  0w, 24w, 48w |
| プロトコールの変更  The change of protocol | 血圧130/80mmHg以上の場合はα-遮断薬、β遮断薬の容量調節可  The regulation of the dose of alpha-adrenergic antagonist and beta-adrenergic antagonist are permitted if the patient’s blood pressure level is over 130/80mmHg. | α-遮断薬、β遮断薬の容量調節不可  The regulation of the dose of alpha-adrenergic antagonist and beta-adrenergic antagonist are not permitted. |

- 研究計画概要（内容）に変更がある場合は、全て提出してください。
- 研究計画に大きな変更がある場合は、申請をし直さなければならない場合があります。
